# Enhancing health care equity with Indigenous populations: evidence-based strategies from an ethnographic study

**DOI:** 10.1186/s12913-016-1707-9

**Published:** 2016-10-04

**Authors:** Annette J. Browne, Colleen Varcoe, Josée Lavoie, Victoria Smye, Sabrina T. Wong, Murry Krause, David Tu, Olive Godwin, Koushambhi Khan, Alycia Fridkin

**Affiliations:** 1 School of Nursing, The University of British Columbia, T201 -- 2211 Wesbrook Mall, Vancouver, British Columbia V6T 2B5 Canada; 2 Manitoba First Nations Centre for Aboriginal Health Research, 715 John Buhler Research Centre, 727 McDermot Ave, Winnipeg, Manitoba R3E 3P5 Canada; 3 Faculty of Health Sciences, University of Ontario Institute of Technology, 2000 Simcoe Street North, Science building, Room 3000, Oshawa, Ontario L1H 7 K4 Canada; 4 School of Nursing and the Centre for Health Services and Policy Research, The University of British Columbia, T201 -- 2211 Wesbrook Mall, Vancouver, British Columbia V6T 2B5 Canada; 5 Central Interior Native Health Society, 365 George Street, Prince George, British Columbia V2L 1R4 Canada; 6 Department of Family Practice, The University of British Columbia, 5950 University Boulevard, Vancouver, V6T 1Z3 British Columbia Canada; 7 Prince George Division of Family Practice, 1302 7 Ave, Prince George, British Columbia V2L 3P1 Canada; 8 Indigenous Health Program, Provincial Health Services Authority of British Columbia, 201-601 West Broadway, Vancouver, British Columbia V5Z 4C2 Canada

**Keywords:** Indigenous people, Health services, Health equity, Health disparities, Canada, Racism, Discrimination, Cultural safety, Structural violence, Trauma informed care, Trauma- and violence-informed care

## Abstract

**Background:**

Structural violence shapes the health of Indigenous peoples globally, and is deeply embedded in history, individual and institutional racism, and inequitable social policies and practices. Many Indigenous communities have flourished, however, the impact of colonialism continues to have profound health effects for Indigenous peoples in Canada and internationally. Despite increasing evidence of health status inequities affecting Indigenous populations, health services often fail to address health and social inequities as routine aspects of health care delivery. In this paper, we discuss an evidence-based framework and specific strategies for promoting health care equity for Indigenous populations.

**Methods:**

Using an ethnographic design and mixed methods, this study was conducted at two Urban Aboriginal Health Centres located in two inner cities in Canada, which serve a combined patient population of 5,500. Data collection included in-depth interviews with a total of 114 patients and staff (*n* = 73 patients; *n* = 41 staff), and over 900 h of participant observation focused on staff members’ interactions and patterns of relating with patients.

**Results:**

Four key dimensions of equity-oriented health services are foundational to supporting the health and well-being of Indigenous peoples: inequity-responsive care, culturally safe care, trauma- and violence-informed care, and contextually tailored care. Partnerships with Indigenous leaders, agencies, and communities are required to operationalize and tailor these key dimensions to local contexts. We discuss 10 strategies that intersect to optimize effectiveness of health care services for Indigenous peoples, and provide examples of how they can be implemented in a variety of health care settings.

**Conclusions:**

While the key dimensions of equity-oriented care and 10 strategies may be most optimally operationalized in the context of interdisciplinary teamwork, they also serve as health equity guidelines for organizations and providers working in various settings, including individual primary care practices.

These strategies provide a basis for organizational-level interventions to promote the provision of more equitable, responsive, and respectful PHC services for Indigenous populations. Given the similarities in colonizing processes and Indigenous peoples’ experiences of such processes in many countries, these strategies have international applicability.

## Background

Despite Canada’s commitment to primary health care (PHC)^1^ and principles of social justice, health inequities remain a pressing national concern [1]. In Canada and other nations, PHC renewal continues to be identified as a key pathway to achieve health equity, with particular implications for marginalized^2^ populations. Substantial evidence shows that PHC enhancements can lead to improved population health outcomes, including reduced acute and chronic conditions, reduced use of emergency services, shorter hospital stays, and lower overall health care utilization [2–5].

In a prior publication [6], we identified four key dimensions of equity-oriented PHC services, which provide a framework for understanding the essential elements of promoting equity among marginalized populations: trauma- and violence-informed care, culturally competent care, contextually tailored care, and inequity-responsive care. Since that publication, our analysis has focused more explicitly on the effects of structural violence and inequities on Indigenous peoples’^3^ health. This paper presents our refined description of the key dimensions of equity-oriented care and specific strategies to use in PHC settings to promote health equity with Indigenous peoples. Using research at two long-established Indigenous health centres in Canada, we illustrate the relevance of these strategies for PHC practices, agencies, and organizations. Although the research we report on in this paper was conducted in the Canadian context, the health of Indigenous peoples throughout the world is shaped by common global colonial and neocolonial forces. Given the similarities in colonizing processes and Indigenous peoples’ experiences of such processes in many countries, these strategies will have applicability internationally.

### Indigenous peoples’ health

Many Indigenous communities have flourished despite colonial forces, however, ongoing social inequities continue to have negative health effects for Indigenous peoples [7–15]. Structural violence is increasingly understood in population and public health as a major determinant of the distribution and outcomes of social and health inequities. Structural violence refers to the disadvantage and suffering that stems from the creation and perpetuation of structures, policies and institutional practices that are innately unjust; because systemic exclusion and disadvantage are built into everyday social patterns and institutional processes, structural violence creates the conditions which sustain the proliferation of health and social inequities [16]. Structural violence can be “static, insidious, silent, taken-for-granted, and hidden” [17] (p. 258), leading many to “interpret disparities in health and income and good fortune as ‘the way things are’" [18] (p. 5).

Structural violence shapes the health of Indigenous peoples and communities globally, and is deeply embedded in history, individual and institutional racism, and inequitable social policies and practices. These continue to exert their effects in the present. For example, in Canada, both the creation of reserve lands with resources inadequate for sustenance, and the apprehension of Indigenous children by the state, initially to residential schools and subsequently to foster homes, have had profound detrimental effects over multiple generations [10, 11, 19]. In Australia, similar attempts at assimilation were implemented through the forced removal of children from Aboriginal and Torres Strait Islander communities. Across colonial contexts, these forced removals are often referred to as  the ‘stolen generation’, and evidence of the harmful health and social impacts continues to mount [14]. Today, the legacy of colonialism, and systemic racism and other forms of discrimination,  contribute to the current lack of employment opportunities, limited access to education, inadequate housing, and high levels of poverty experienced by many Indigenous peoples in Canada, Australia, and other colonized countries throughout the world [10, 20, 21].

### Gaps in health services: the need for strategies to address the health effects of structural violence

The historical and ongoing forms of structural violence experienced by Indigenous peoples have unfolded against the broader context of neoliberal economic reforms and social spending cuts over the past three decades. Canada has become second only to the USA in its growing levels of income inequality [22]. The lack of affordable housing in many parts of Canada, and the loss of community-based social and health services disproportionately burden Indigenous peoples. Within this context, the health and wellbeing of Indigenous peoples continues to lag behind that of the overall Canadian population on virtually every measure [7, 9, 10, 20, 23–27]. For example, it is estimated that by 2017, the mean life expectancy of Indigenous men will be 70.3 years compared to 79 years for all Canadian men, and 77 for Indigenous women compared to 83 for all Canadian women [28]. Throughout Canada, infant mortality rates among Indigenous populations are dramatically higher compared to other Canadians [23, 29]. Today, Indigenous children represent an alarmingly high percentage of the children in government care^4^, reflecting the current state surveillance of Indigenous women, families, and communities [30, 31]. Research also shows that the high rates of HIV infection among Indigenous women [32–34] and the disproportionate likelihood of violence against Indigenous women and girls are explained by the unique experiences of colonization of Indigenous peoples in Canada, founded on ongoing racism and discrimination [9–11, 35–38].

Internationally, the traumatic, negative impacts of historical and ongoing colonialism on Indigenous peoples’ health and well-being are immense. Globally, Indigenous peoples experience significantly higher rates of ill health and have dramatically shorter life expectancies than other groups living in the same countries [39, 40]. In Australia, life expectancy at birth for Indigenous peoples is estimated to be 19–21 years lower than their non-Indigenous counterparts [40]. In parts of Central and South America, infant mortality rates are extremely high for Indigenous peoples, reflecting limited economic opportunities, poor access to social services, and high levels of public insecurity [40]. Throughout the world, Indigenous peoples experience disproportionately high levels of maternal and infant mortality, malnutrition, cardiovascular illnesses, HIV/AIDS and other infectious diseases, such as malaria and tuberculosis [39]. These morbidity and mortality patterns are strongly connected to histories of colonization, the dispossession of lands and cultural and economic resources, and the ongoing lack of access to the social determinants of health. As emphasized in the report of the United Nations Special Rapporteur, these health status disparities are compounded by the persistent and multifaceted forms of racial discrimination experienced by Indigenous peoples globally: “such discrimination is intimately interconnected and mutually reinforcing with the spectrum of violations experienced by Indigenous peoples” [39] (p. 10). Research in Australia, for example, continues to show that ineffective, insensitive and inappropriate care contributes to Aboriginal peoples’ experiences of shaming, misunderstanding and stereotyping [41].

Discrimination in health care is continuous with experiences of discrimination in people’s everyday lives. Racial discrimination is further amplified in the contexts of poverty, substance use, or stigmatizing conditions such as chronic pain, mental health issues, and HIV [42–50]. Research confirms that Indigenous peoples experience individual and systemic discrimination when seeking health care [6, 11, 27, 44, 51–53], despite efforts within the health care sector to promote cultural sensitivity and cultural safety. For example, despite long-standing evidence of lower per capita alcohol consumption by Indigenous peoples compared with the general population, one of the most common public perceptions in Canada reflects racialized assumptions about Indigenous peoples being prone to alcohol or substance use [11, 54]. The colonizing image of the ‘drunk Indian’ is one of the most harmful stereotypes operating in health care settings [55]. These assumptions intersect with messages in the media, public venues and everyday conversations in Canada to undermine Indigenous land claims, rights, and entitlements, and in health care contexts, shape decisions about which patients are credible and deserving of care [6, 44, 52, 53, 56]. Thus racism and discrimination must be considered determinants of health for Indigenous peoples, and strategies are required to mitigate the negative impacts on health [11, 25].

Health services, however, are not typically designed to take into account the experiences of Indigenous peoples [10, 25, 57, 58]. For example, despite extensive evidence linking trauma and violence to multiple health problems, including chronic pain, depression, anxiety and substance use [57, 59–62], these dynamics are rarely considered in the design and delivery of health care for Indigenous peoples [7, 63–69]. A decolonizing lens is useful for addressing this complex interplay of factors [66, 70, 71], by directing attention to the root causes of people’s health and social issues. In this paper, we offer a framework and specific strategies for promoting equity-oriented care that takes into account the colonial history and ongoing subjugation of Indigenous peoples, and that supports Indigenous peoples’ agency and resistance to such subjugation.

## Methods

The findings discussed in this paper resulted from a larger study that: (a) examined how PHC services are provided to address the health needs of Indigenous and non-Indigenous peoples experiencing the health effects of systemic inequities; (b) identified the key dimensions of equity-oriented PHC services for marginalized populations; and (c) developed a set of PHC indicators to account for the quality, process, and outcomes of care when marginalized populations are explicitly targeted. This four-year study used a mixed-methods, ethnographic design and was conducted in partnership with two urban Aboriginal health centres located in two inner cities in Canada. The research team was guided by an Indigenous community advisory committee (CAC) including leaders in Indigenous health services, patient representatives, and people recognized as Indigenous Elders. The CAC was regularly consulted about methods, the interpretation of research findings, and strategies for discussing the implications with Indigenous peoples and health care leaders. All aspects of our research process received ethics approval by two ethics review boards.

This study was informed by critical theoretical perspectives and Indigenous epistemologies. Critical theories are useful for drawing attention to the political and moral concerns arising from the legacy of colonialism, and how this shapes people's everyday experiences [72–74]. A central methodological concern is that individual experiences, including those in health care, need to be interpreted and understood within the context of broader social, political, and historical relations. Indigenous epistemologies provide a broad vantage point from which to understand the complex “web of relations” [75] that are encountered in health care. Although Indigenous epistemologies encompass a range of diverse ideas, they converge on the idea that knowledge is underpinned by a world view that reflects interconnectedness, relational values, and the pursuit of knowledge about relationships among people, the land, and community [76–78]. Critical perspectives and Indigenous scholarship are committed to challenging Eurocentric assumptions and value structures in both academia and society at large.

A detailed description of the research methods is provided in an earlier publication [6] and summarized here. The two health centres that served as sites for this study were established in the early 1990s in western Canada as urban PHC clinics with an explicit mandate to provide interdisciplinary team-based services to Indigenous and non-Indigenous peoples experiencing significant health and social inequities. The clinics serve a combined patient population of 5,500, the majority of whom identify as Indigenous. Many of the patients live on less than $1,000/month (far below Canada’s poverty line), and due to a lack of affordable housing, live in unstable or unsafe housing, or in shelters or single-room occupancy hotels. A high proportion have histories that include adverse childhood experiences [79–81] (including, for example, being removed by the state from the care of their parents, child abuse of all forms, and the death of family members), and many face interpersonal and structural violence in their everyday lives as adults, including ongoing discrimination and stigma related to systemic racism, mental health issues, and substance use. To respond to people’s overlapping health and social needs, both clinics provide team-based care by nurses, physicians, social workers, counsellors, outreach workers, and pharmacists, among others, and to varying degrees, access to Indigenous Elders who provide support for both Indigenous and non-Indigenous patients.

Data were collected by the principal investigators during intensive immersion at the clinics. We conducted in-depth interviews with a total of 114 patients and staff (*n* = 73 patients; *n* = 41 staff), including: (a) individual interviews with 62 patients, and three focus groups with a total of 11 patients; and (b) individual interviews with 33 staff, and an additional eight staff who participated in focus groups. We conducted over 900 h of intensive participant observation focused on staff members’ interactions and patterns of relating with patients and other staff during clinical encounters and in waiting rooms, staff meetings and case-management discussions.

Of the patients who participated (*n* = 73), 52 % were women, 45 % were men, and 3 % identified as transgender. Seventy-seven percent self-identified as Indigenous, 22 % as Euro-Canadian, and 1 % as members of a visible minority^5^ [82]. Ages ranged from 20 to 72 (mean = 45 years). Of the clinics' staff who participated (*n* = 41), 24 % were nurses or nurse practitioners, 22 % were physicians, 22 % were medical office assistants and office managers, 10 % were in administrative leadership positions, 7 % were social workers, 5 % were substance use counsellors, and 10 % were other staff including an Elder, an outreach worker, a support worker, and a pharmacist.

We conducted an interpretative thematic analysis [83] using NVivo to assist with organizing and coding the interview and observational data. As data were collected and analyzed, coding categories were refined. In the final stages, the analysis shifted to a more abstract and conceptual representation of themes and key dimensions of equity-oriented care. The credibility of our analysis, as a criterion for rigour in qualitative research, was assessed through regular meetings with the Indigenous CAC and discussion sessions with patients at both clinics. These stakeholders confirmed that the identified themes reflected their experiences and interpretations, and that the framework and strategies we propose capture those features of PHC services that are necessary to optimize care in partnership with Indigenous populations.

## Results and discussion

**Fig. 1:**
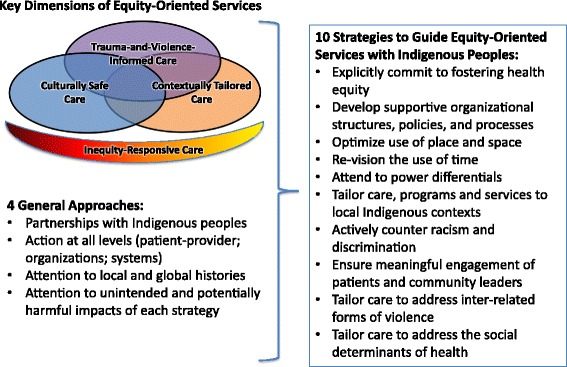
Essential Elements of Equity-Oriented Primary Health Care with Indigenous Peoples

We previously identified key dimensions of equity-oriented PHC services which are particularly relevant when working with diverse groups of marginalized populations [6]. In the case of Indigenous peoples, these key dimensions need to be conceptualized in ways that take into account the historical and ongoing forms of discrimination and structural violence that continue to shape Indigenous peoples’ health, well-being, and access to resources. In Fig. 1, the four key dimensions are operationalized using four general approaches and 10 strategies that intersect to optimize the effectiveness of services. These can be locally tailored in partnership with Indigenous communities.

### Key dimensions of equity-oriented PHC revisited

As shown in Fig. 1, inequity-responsive care is foundational to supporting health and well-being, and requires explicit attention to the provision of culturally safe care, trauma- and violence-informed care, and contextually tailored care. These are explained briefly below:

#### Culturally safe care

Cultural safety was originally developed in New Zealand by Māori nurse leaders in consultation with Māori communities as a pragmatic tool for moving nursing and health care practices beyond the notion of cultural sensitivity to more actively address inequitable power relations, discrimination and racism, and the ongoing impacts of historical injustices on health and health care [84]. Cultural safety has the potential for shaping health care practices, organizations, and policies by identifying social justice goals as integral to health care, and by shifting attention away from cultural differences as the source of the ‘problem’ and onto the culture of health care as the site for transformation. Increasingly in Canada, the USA and Australia, cultural safety is featured as an essential element of health care involving Indigenous peoples [85–87]. In New Zealand, cultural safety is legislated as a basic requirement of nursing and medical professional education [88].

#### Trauma- and violence-informed care (TVIC)

The concept of trauma is used increasingly to inform care provided to people who are marginalized by social and structural inequities [57, 61, 62, 89, 90]. The term trauma can be problematic in part because it signifies both traumatic events (often presumed to have occurred only in the past) and the responses to such events (often presumed to be only psychological). Indigenous and non-Indigenous scholars [62] critique this ‘trauma trend’ because it both obscures the impact of ongoing structural violence and is often used to pathologize Indigenous peoples. We share these concerns and endorse the call for using a decolonizing lens when discussing trauma in relation to Indigenous peoples [62]. Integrating attention to violence when discussing trauma keeps the focus on violence (both historic and ongoing), and reduces the likelihood of locating the ‘problem’ only in relation to the psychological impacts for those who have experienced violence, rather than also on structural violence and the conditions that support it [62].

Our understanding of TVIC draws on previous work on trauma-informed care, but is founded on the assumption that people disadvantaged by systemic inequities experience varying forms of violence that have traumatic impacts on an ongoing basis. These impacts include a wide range of symptoms and health problems such as chronic pain, mental health issues and substance use. TVIC involves providing care that is respectful and affirming, and requires all staff within any given organization to (a) recognize the intersecting health effects of structural and individual violence, and other forms of inequity; (b) understand people’s health and social issues in context; and (c) work to reduce re-traumatization. Importantly, TVIC is not about eliciting trauma histories; rather, the goal is to create a safe environment for all based on an understanding of the traumatic effects of historical and ongoing violence and discrimination.

#### Contextually tailored care

Expanding the concept of patient- and family-centred care, contextually tailored care includes services that are explicitly tailored to the local communities and populations served. This may include tailoring practices and/or organizational policies and clinical guidelines to address the needs of local population demographics, and social and community realities that often shift depending on local politics, epidemiological trends, and economic conditions. For example, depending on need, some populations might benefit from mental health support for families dealing with the intergenerational effects of residential schools, support for women raising young children, or home visits for isolated seniors. Individuals working in health care cannot enact these strategies without supportive organizational strategies and policies, or without an awareness of the multiple contexts that shape both Indigenous peoples’ health and the broader sociopolitical environment in which health care is provided. We emphasize the following four general approaches as a basis from which to enact strategies to operationalize the key dimensions of equity-oriented care for Indigenous populations.

### Four general approaches

#### Develop partnerships with Indigenous peoples

Partnerships with Indigenous peoples and/or leaders, agencies, and communities are required to operationalize and tailor these key dimensions to local contexts. These partnerships can begin in small ways and develop over time [91, 92]. For example, in Canada, partnerships could be developed through consultations with local Elders, Friendship Centre^6^ staff, local First Nations, Metis and Inuit organizations, and/or by structuring Boards or other governance bodies to include Indigenous peoples or representatives.

#### Take action at all levels

The practices of individual health care providers are important, but insufficient to achieving health equity. Action is required (a) at the intrapersonal level among all levels of staff involved in health care organizations and systems, with an emphasis on people's values, beliefs and assumptions, and their capacity for reflexivity; (b) at the interpersonal level to optimize the interactions among providers and patients, among staff, and between different health and social service organizations; and (c) at the contextual level, including efforts by staff, managers, and leaders to affect change within health care organizations and the wider community.

#### Attend to local and global histories

While the approaches and strategies in this paper are intended to promote equity in PHC for Indigenous peoples, we are explicitly not advocating a pan-Indigenous understanding. That is, in full recognition of the diversity of Indigenous peoples, an understanding of local history is essential. Although Indigenous peoples may have some shared experiences of historical and ongoing colonization, these histories are diverse, as are the cultures, languages, and traditions of the people involved. Promoting health equity requires understanding the erosion of Indigenous peoples’ power and resources as purposeful in the service of historical and contemporary colonial conquest.

#### Attend to the unintended and potentially harmful consequences of each strategy

We emphasize caution to pre-empt potentially harmful uptake of the 10 strategies outlined below, given predominant assumptions, policies, and practices that currently drive health care systems. Hence, we identify how to avoid, for example, the potential for culturalism^7^ or essentialism to seep into the interpretation of these strategies. Describing what the strategy ‘is not’ is particularly important in today’s health care climate where decisions about efficiencies and performance management may have serious implications for deepening, rather than ameliorating, health inequities among Indigenous populations.

### Ten strategies to guide equity-oriented services with Indigenous peoples

Using illustrative examples from our datasets, the 10 strategies are discussed in relation to health care involving Indigenous peoples. Depending on the type of PHC organization involved (e.g., individual physician practices, or team-based agencies offering wrap-around services), aspects of these 10 strategies will be more relevant or more feasible at particular points in time. Consistent with interpretive inquiry, literature is integrated into the discussion of findings to form linkages among the empirical data and relevant theoretical perspectives.

#### 1. Explicitly commit to fostering health equity in partnership with Indigenous peoples in mission, vision, or other foundational policy statements

Operationalizing an organization’s or unit’s commitment to health equity begins with attention to values, and an intention to work toward shared values at the level of individual health care providers, and in relation to organizational structures, policies, and processes. As emphasized by this physician about the clinic in which he worked: “It’s a bigger place for values, shared values… We do try and understand, you know, the historical context. We try and understand where people are coming from… the things that are impacting their health”.

Throughout Canada, health care organizations serve diverse communities and populations. We have argued elsewhere that it is important for health care organizations to develop strategies for fostering health equity in ways that will result in overall improvements in care [6, 42, 44]. However, in Canada, because the most extreme health inequities persist in relation to Indigenous peoples, and because racism toward Indigenous peoples is so pervasive, it is morally imperative to articulate an explicit commitment to foster health equity in relation to Indigenous peoples in mission or vision statements and in documents outlining strategic directions or aims. As emphasized above, partnerships with Indigenous peoples are integral to this strategy, and as we discuss further, the strategies have the potential to positively influence health services more widely.

#### 2. Develop organizational structures, policies, and processes to support the commitment to health equity

While setting the intention is a first step, supportive infrastructure is required. Individual health care providers, managers, and funders must be supportive of the structures, policies, and processes that will foster equity for Indigenous peoples through the 10 strategies. This means every staff member must examine their values, assumptions, and privileges. Structures, policies, and processes related to hiring, performance evaluation, recognition, rewards and compensation, continuing education, and staff meetings all must be viewed with respect to equity. For example, staff whose values align with the commitment to equity must be recruited, hired and retained. This means that organizations need to invest time to recruit both Indigenous and non-Indigenous staff members whose values and motivations for working with Indigenous populations align with the social justice orientation underlying the key dimensions of equity-oriented care and the 10 strategies. Given health human resource shortages, and the relatively few nurses and physicians who are of Indigenous descent in the Canadian workforce, it remains challenging to recruit staff members who identify as Indigenous. As one First Nations staff member in our study described, although the goal is to hire Indigenous staff, it is not always possible; however, it is critical that staff demonstrate a commitment to values that align with a social justice and health equity mandate:

It doesn’t always have to be Aboriginal people that run an Aboriginal agency. The values just have to be there entrenched in the policies, being able to look at service delivery, those kinds of things… it can be very difficult if somebody comes to work here who may not have the same values.

Further, staff whose commitments are not aligned cannot be retained. In both clinics, staff members were asked to leave, or decided to leave with the remaining staff expressing consensus that there was a mismatch between the values of the organization and the particular person. This can be challenging in both unionized and non-unionized environments for different reasons, including both legal reasons and community opinions. For example, employees at the clinics who were well-known in the wider community were asked to leave because of mismatches, but the reasons for dismissal could not be shared, causing tensions. In some cases  considerable human resource and legal work was required to affect dismissal.

Importantly, staff members whose values *do* align with those of the organization must be supported to work effectively in their roles. Organizational supports, including access to support for vicarious trauma, are needed for all staff working with patients who experience the effects of structural violence on a daily basis. It is particularly essential for organizations to implement strategies to support Indigenous health care providers and other staff who have also been subjected to structural or other forms of violence and trauma in their lives, and who are caring for patients exposed to such trauma. In this study, staff members at both clinics provided care to people experiencing extreme forms of structural violence, but without adequate support for dealing with vicarious trauma, as this program manager emphasized:

In the last fourteen years we’ve lost about twenty Aboriginal staff…. And, of course, we don’t have any type of psychology services or EAP [employee assistance program]. What we have are each other… we can’t just hire Aboriginal people and not realize how this workplace can re-trigger… and re-traumatize… I’m saying we need experienced people. We make it a policy to hire Aboriginal people however when we hire people, we can’t just hire on the basis of being Aboriginal. You can’t just throw a new grad in and say “go to work”. You can’t.

#### 3. Optimize use of place and space to create a welcoming milieu

Underpinning all 10 strategies is a commitment to creating spaces and places where people feel welcome when they come for health care, as described by this patient:

You don’t have to sit in a room, like in a doctor’s office, and be real square, you know, be really uptight. Here, it's like you see people walking back and forth, conversations happening all the time. It's like you’re a piece of this place, you're not just a number. It’s like a home.

This is especially significant given the patterns of dismissal that many Indigenous peoples experience. As Wendt and Gone [93] and others [53, 94] have argued, when people experience dismissal, invisibility, or conversely, are hyper-visible, it is especially important to create settings in which people feel they are deserving, understood, recognized, and accepted. As found in Wendt and Gone's [93] study of urban-Indigenous therapeutic landscapes, patients at the clinics reiterated their view of these places as crucial urban spaces often identified as home:

Like at other places, you don’t have a personal relationship with the staff… Here, like, you chat with the staff and they offer support for other things in your life that you need. It's more personal here.

#### 4. Re-vision the use of time

Re-visioning the use of time in providing care is an essential feature of equity-oriented care. Flexibility is required to foster trust with people who are often dismissed or mistreated within the health care system. For example, in this study, both clinics’ scheduling systems were continuously revised (balancing drop-in with scheduled visits) to be as responsive as possible to what patients perceived as their highest priorities, as this woman described:

If we’re in a bad situation, we can more likely just pop in to see [one of the staff]. You know, and get it out of your system. It’s good to know that there’re people here that will actually listen to you.

Developing and allocating time for follow-up actions for those who might otherwise ‘fall through the cracks’ is especially important for patients whose social support systems are limited. This conveys to patients that staff are concerned about their well-being beyond the boundaries of the clinic’s physical space. This woman, a patient at one of the clinics, described the significance of such follow-up:

They record if I don’t come to the doctor, so that makes me feel good because, uh [pause], because I know that if something should happen to me outside of the clinic, and if I don’t come here, they’re going to definitely acknowledge that. Because they know how important to me my health is… and so they would definitely try to track me down through my family and so on… So, that’s one main reason that I come here.

In the context of providing care to Indigenous peoples, re-visioning the use of time is not founded on culturalist assumptions about time being differently understood by Indigenous peoples. Rather, this is a strategy and means of providing services that are consistent with the key dimensions of equity-oriented health care. Taking the time required to provide good care is important, not as a ‘cultural’ value, but because colonial processes have been and continue to be so harmful. Re-visioning time does not necessarily require spending *more* time with people. Rather, time is used differently, for example, to validate and recognize that people are making their best efforts to deal with often overwhelming situations and environments. Health and healing are recognized as life-long processes that require providers to develop relationships with patients over time. Whereas in some health care settings, patients are ‘banned’ or ‘fired’ when they do not adhere to recommended treatments or advice, understanding the effects of structural violence and discrimination leads providers committed to equity to never ‘give up’ on or abandon patients.

Fostering genuine partnerships with communities or community leaders, and seeking their input into programs or policies, also requires flexible use of time to ensure meaningful involvement of all stakeholders [95]. Performance indicators and quality assurance measures are typically not designed to account for these kinds of investments in time. At both clinics, administrators and staff were frequently required by the Health Authority and funders to justify their regular interdisciplinary team meetings, which were essential to engaging in team-based case management, developing consistent approaches to care, and forming community partnerships. This highlights the need for measures and indicators that can capture the value of these activities, so that these aspects of service delivery are legitimized and made visible as essential components of equity-oriented health care [96, 97].

#### 5. Continuously attend to power differentials

Given the historical and ongoing abuses of power toward Indigenous peoples in Canadian society and in health care, underpinning all other strategies is the investment required at all levels of the organization to continuously attend to power differentials. Working to mitigate power differentials requires attention to language and discourses in health care that sustain  inequities. For example, given the pervasiveness of individualist ideology in health care, and how often individualism is used as a framework to blame Indigenous peoples for their circumstances, health care staff need to actively develop diverse strategies to mitigate the myriad ways that judgments about ‘personal responsibility’ are conveyed in verbal and non-verbal interactions with patients. For example, providers at both clinics described how they had learned to respond to people in their professional and social networks who expressed judgements about Indigenous peoples as being responsible for their own suffering or having some genetic predisposition to health or social problems (such as substance use). Similarly, egalitarian discourses that advocate ‘treating everyone the same’ de-historicize and de-politicize the complex factors that underpin social suffering, and divert attention away from the structural inequities that influence health and access to health care [52]. Attending to power differentials in the context of providing health care to Indigenous peoples requires recognizing that the dominant ideological doctrine of ‘treating everyone the same’ can actually reproduce inequities by blinding health care providers to their relative privilege and biases, to the unequal power relationships between patients and providers, and to the social inequities that differentially shape people’s health. As these staff members explained:

So, it’s almost like you’re counteracting that. Like, I’m counteracting those things as a professional. And to be able to be aware of the reality of somebody who lives in the core [inner city] area, who is poor… I want to make this person feel that even though I’m a [provider], I am at the same level as they are. I don’t place myself above them or anything like that. There is no status of power when I work with people. I try to keep that as minimal as possible.

We try as much as possible, I believe, to be not as judgmental. The way people, the patients, have told us, how they feel when they go to emergency or the other government bodies is that they feel kind of judged and discriminated against. I think we are all here to try to be non-judgmental and to treat everybody with respect and, you know, I think that pays off.

People who experience inequities and marginalization often experience dismissal and/or stigma when accessing health care or community services. As research continues to show, such power inequities are frequently magnified for Indigenous peoples in Canada [6, 30, 44, 53, 98]. Experiences of discrimination and dismissal, and the consequent lack of trust in health care systems, underpin people’s efforts to avoid contact with the health care sector. Health care providers and organizations need to anticipate and expect that many people, based on their own and others’ histories, will feel mistrustful of the health system’s ability to offer help, with further isolating effects. As one of the patients said of herself and others attending the clinic: “They don’t trust, and to gain their trust is to… kind of listen to them [patients], and it will come back”.

The significance of earning patients’ trust in the face of long-standing experiences of mistrust was described by another woman, who discussed her process of working with staff as she tapered herself off what she perceived to be an unhealthy, long-term dependence on benzodiazepines:

I’ve encountered so many people here [at the clinic], and nobody lies to me… But you know what: if it wasn’t for this place, I wouldn’t have been who I am today. Seriously. That is not a lie… They helped me find myself… They helped me with the trust. And a lot of people can’t trust a clinic, you know.

Attending to power differentials requires all staff to realize that how one is perceived as a member of the health care system cannot be separated from one’s position in broader socio-historical relations. Even if a staff member does not intend to act in a ‘power over’ or discriminatory manner, his or her social location as a member of the health care system can be seen by patients to imply that power inequities will be maintained and shape their access to services [52].

Countering power differentials in organizations involves adapting clinic procedures in ways that convey respect and preserve people's dignity. For example, both clinics explicitly decided not to staff waiting rooms with security guards. Rather, patients' expressions of anger or frustration were met with respectful responses, especially by medical office assistants. Our field notes reflected the relatively rare occurrences of aggressive or violent behaviour in the clinics in comparison to our field work observations in Emergency Units, where security guards were present.

#### 6. Tailor care, programs, and services to local contexts, Indigenous cultures, and knowledge systems

Experiences of colonization have important similarities for many Indigenous peoples, and these similarities are the basis for many of the proposed strategies; however, the diverse and particular histories, cultures, and knowledge systems of the people served must be used as the foundation for tailoring care. This requires all staff (and board members if applicable) to learn about the specific pre-colonial and colonial histories of local communities. This can be supported by managers and leaders through staff orientation and continuing education, and by integrating Indigenous knowledges, languages and concepts into care. These efforts can be promoted through partnerships with knowledge keepers, Elders, and other Indigenous leaders. This approach is in direct opposition to the more common efforts by health care organizations to address the needs of Indigenous peoples through ‘cultural sensitivity training’. Although attention to culture is important, this is often done in isolation from other fundamental concepts and frameworks, such as social determinants of health, advocacy, and social justice [99, 100]. Cultural sensitivity training can also reinforce potentially stigmatizing notions of Indigenous identity, inadvertently resulting in ‘othering’ practices [99, 100]. Health care providers and organizations working with Indigenous populations, therefore, need to guard against the culturalist tendencies pervasive in health care, which reinforce popularized, stereotyped notions of culture as the primary analytical lens for understanding people’s health issues. For example, both clinics have worked hard to integrate culture into their health services, and regularly integrate smudging^8^ and teachings by Elders, as described by this staff member:

We have Elders here… and we have the smudge ceremony during our meetings… At one point someone [would] come in twice a week to do smudges with the clients and they would do the drum and sit out there [in the waiting room] and… would talk to people… There was much more of a calmer atmosphere when he was here… It really changed the dynamics of the waiting room… It’s like an innovative idea but it’s actually quite, in some ways I guess, common sense, but you don’t often think of putting someone who’s going to bring some healing words and energy into the waiting room.

These cultural practices are integral to both clinics, but alone are insufficient for equitable care. Rather, tailoring requires meaningful partnerships, integration of understandings of local history, and integration of Indigenous knowledges, as discussed by this physician:

I like to think that I use some of the bigger principles as in… taking the narrative and the stories from people and listening… They’re principles that kind of help me in keeping a more open view of the situation so I don’t get stuck on a medical model… It’s beyond that. It's the spiritual aspect and the family aspect and that historical aspect, because I mean history is every day in the clinic. There’s history that’s hugely significant in the work I’m doing there.

Organizations that are planning to tailor programs to local Indigenous contexts need to be flexible in how funds are allocated [101]. Indigenous knowledge can be integrated in a variety of ways through partnerships in the local community, and the process can have powerful impacts within an organization. These approaches should be locally-determined given that Indigenous knowledge is grounded in local contexts, communities, histories and protocols [102, 103].

#### 7. Actively counter systemic and individual experiences of racism and intersecting forms of discrimination

Given the high proportion of Indigenous peoples in Canada who experience racism and discrimination, counteracting people's experiences of discrimination at an individual level requires that health care providers convey an understanding of history and context. As this patient described, these dynamics routinely shape many Indigenous peoples’ health care experiences:

Toward Native people, you know, if you go to a different organization, you know, you get all the looks, and you know, like, “what are you doing here?”, that kind of service. Just to get some kind of service, you have to really act good.

Conveying knowledge, genuine interest, and willingness to listen to people in an unhurried manner can create space for people to feel more fully understood, as described by this patient:

Like when you go to a regular doctor, they don’t sit and ask you how you’ve been, you know what I mean? So, they know your whole situation. I go to [another doctor] and he’ll just feed me pills.

Repeatedly, staff and patients identified that the impact of such efforts was often powerful. This physician described how supported his patient reported feeling after inquiring about the impact that residential schooling had on the patient's health:

I had discussions about the residential school, and I didn’t remember even ever talking about [it] and hadn’t documented much in the chart. Yet he thought that, or he felt that he’d been incredibly supported in his experiences through residential school and the traumas that he had there. So somehow, whatever he’d brought up, and whatever I did, he felt like he was validated. But I didn’t even think it was significant. So he was getting benefit that I didn’t even acknowledge or realize.

Countering racism and discrimination, which is linked to addressing unequal power relations, demands that health care providers have an acute awareness of how racism intersects with gendered inequities, classism, and other forms of discrimination, as this staff member explained:

Listening to somebody talk about how they went somewhere [within the health care system] and how they waited and how the doctors treated them differently. And then, it could be as simple as saying, “well I’m sorry that happened to you,” being able to validate how they feel rather than saying, “oh well, that’s the system and you have to work through it.” And it’s just really small little things. And it is people coming back and saying, “well, that really helped me.” And you may not even remember saying that or you may not even remember doing those kinds of things.

Organizations that develop strategies to counter racism and discrimination need to push back against dominant, neoliberal discourses that reinforce misconceptions about people having  equal access to health care and resources [52, 53, 98, 104]. The significance of ensuring that people feel welcome in health care spaces cannot be overemphasized. Repeatedly, patients explained the significance:

You see the people that walk in here, I mean, they’re not always nice looking characters. But you’re always welcome. They always treat you like you’re just as good as the next person.

Actively counteracting discrimination in organizations requires that claims of discrimination be considered seriously, regardless of intention. Often patients who express concern about being disrespected are assumed to be overly sensitive, reactive, or attempting to gain unfair advantage by making such claims, and research shows that people who experience discrimination on a daily basis may not be able to distinguish intentional from unintentional unfairness [44, 45, 47]. At the organizational level, it is therefore imperative to provide mandatory, explicit, anti-racist training for staff at all levels, including administrators, managers, receptionists, and direct service providers. Recurring training is required to revisit strategies for countering the persistent potential for Indigenous peoples to be treated in inequitable ways within the health care sector.

#### 8. Ensure opportunities for meaningful engagement of patients and community leaders in strategic planning decisions

As discussed in the first strategy, addressing the root causes of health, social, and health care inequities requires partnerships with Indigenous peoples and community members. Engagement can take many forms, depending on organizational context, governance structures, and patient populations. Patients involved in this study, for example, reiterated the significance of being asked for input or volunteering their time to assist with seemingly mundane tasks related to upkeep of the clinic’s premises. As this patient explained:

When I sit out here [in the clinic’s waiting room], I see different skills of people… So I’m thinking, when I go back to my reserve, I want to give back. Everybody has that. I want to give back. Look at how much I’ve taken from my family. Everyone down here has that feeling. Even when they have nothing they want to give back.

In some settings, the formation of a Community Advisory Committee or an Elders Committee may be instrumental in ensuring that integration of Indigenous knowledge aligns with culturally safe intentions, and to avoid the appropriation of knowledge. In other settings, opportunities for employing patients as peer support workers or volunteers may be feasible, and can have significant impacts on the quality of life for those involved, as this patient described:

I want to help people, I want to do stuff in this world for people and this is one of… why I’m doing this [as a] positive prevention peer support worker.

Finding opportunities for community members to be meaningfully involved in some aspect of the clinic or programming also provides a means for acknowledging and drawing on the strengths, capacity, and agency of community partners.

#### 9. Tailor care, programs, and services to address interrelated forms of violence

Health care providers need to recognize that some Indigenous people may be survivors of multiple forms of violence with traumatic effects, while still experiencing current and ongoing interpersonal violence (including racial violence and intimate partner violence), and ongoing structural violence (such as systemic and organizational racism, absolute poverty, etc.) [11, 105–107]. Tailoring care, therefore, means offering comprehensive care that simultaneously addresses the multiple consequences of interrelated forms of violence.

TVIC is one way of respectfully tailoring care to the impact of history, specifically histories of violence, on people’s lives. At the individual level, this includes acknowledging the impact of social injustices and structural violence on people's sense of agency, that is, the constraints and possibilities for health and well-being in any individual's life. Thus, the strategy of re-visioning time intersects with this strategy in that the ‘pace’ of healing will be influenced by each individual’s history. At the organizational and provider levels, tailoring to address interrelated forms of violence involves developing strategies to acknowledge that dispossession from Indigenous lands, territories, cultures and languages is a fundamental determinant of health and form of structural violence that is often at the root of people's mental health and substance use issues [21, 108].

Research shows that organizations informed by understandings of TVIC recognize that issues of substance use, chronic pain, and histories of trauma are integrally interconnected for people experiencing structural violence [11, 25, 37, 58, 89, 105, 109–111]. Such organizations also recognize that people who present with these intertwining issues frequently mistrust health care services. Therefore, for example, services must simultaneously address substance use, and the health consequences of violence and trauma, including post-traumatic stress disorder, chronic pain, and sleep problems according to the patients' priorities. As in most PHC settings, pain was one of the most common presenting concerns among patients in this study, as this provider explained:

Managing pain in this environment is exceedingly challenging because people do have emotional pain and they do have physical pain and there is a gray area and they overlap.

Addressing the consequences of interrelated forms of violence includes being responsive to the multi-faceted nature of pain, and developing strategies for addressing pain from a trauma- and violence-informed perspective. Similarly, substance use issues must be addressed by taking chronic pain and trauma histories into account. This requires adapting conventional guidelines for treating chronic pain, mental health issuses, and substance use in ways that recognize the effects of trauma and violence on health, and that extend beyond the often tense negotiations for prescription drugs.

Elsewhere we have described the experiences of Indigenous peoples who express concerns about being viewed as ‘drug seeking’ when they were seeking help for what they perceived to be legitimate pain issues [44, 53, 112]. Similarly, in this study patients argued for:

… a lot more doctors with compassion for Aboriginal people, instead of just looking at them like they’re drug addicts or alcoholics, and not helping with the real pain, which all of us do have real pain.

Without a broader understanding of the intertwining nature of trauma, pain, and substance use, negative judgments conveyed to patients, particularly those who experience problematic substance use, can have harmful consequences, as this patient described:

The whole thing of addiction is having people listen, and not judging. And most doctors I know, except for the select few that are here, they are all judging, very judgmental of addicts [sic]. Especially at the hospital. I would rather go through severe pain than go to the hospital.

At an organizational level, training about providing care for patients who have experienced violence, and the intersecting health and social impacts, has been shown to increase clinicians’ confidence, knowledge, and efficacy [113–115]. Such training is essential to integrate understandings of the long-term impact of trauma into practices and policies, and to avoid inadvertently creating an invalidating environment [61, 90, 116]. This provider explained the growing integration of TVIC at their PHC clinic:

… critically thinking how we provide care and always going back to those core things of where that person has come from. So now when we talk [at staff meetings], we don’t just talk about [patients] needing this or that, we go to a much deeper level. It’s very interesting to see the switch that’s happened with staff because they’re now talking about, you know, that’s really re-traumatizing or do you realize the level of trauma this person comes from? Or do you realize how difficult it is for that person to walk through that door? Or do you realize how difficult it was for that person to actually bring up their concern to you?… And so we, we now are getting into conversations around our table about power imbalances and about how our interactions can really affect this. So I’ve seen our place come from being just a place of, you know, a safe place to come to, but it’s now come to a place where I can see staff grasping these ideas and talking to each other.

Given the high rates of state apprehensions of Indigenous children [10] and growing evidence of the benefits of supporting women to care for their own children [117, 118], it is critical for PHC organizations and individual providers to develop strategies for building trusting relationships with women and families. Strategies include working with social workers and other team members to support parents’ access to visitations with children removed from their care. At an organizational level, these kinds of efforts could be framed as part of a larger strategy to support both men and women. PHC providers can contribute to interrupting the intergenerational effects of residential school by recognizing the continuities between residential school and contemporary child protection practices, and supporting Indigenous peoples to parent their own children. For example, in one of the clinics, support for pregnant and post-partum women who were under surveillance by state authorities involved working in partnership with child protection workers, social workers, and other social services staff to develop shared-case management plans.

#### 10. Tailor care to address the social determinants of health for Indigenous peoples

Increasing access to the social determinants of health is important for all people who experience health inequities. Due to welfare state colonialism, economic constraints on reserves, and discriminatory policies, this strategy requires intensive effort in relation to Indigenous peoples.

At a provider level, health issues must be continually understood and addressed within the context of the social determinants and how they are specifically constrained by myriad colonial policies for Indigenous peoples. For example, access to stable housing is constrained by the consequences of the reserve system in Canada, by shifting policies that determine who has ‘status’ and by discrimination in housing, employment, and education. As this physician explained, the social determinants of inequities for Indigenous peoples must continuously inform clinical care:

It does shape diagnostic and therapeutic decisions… If their housing is unstable… those kinds of things are all very pertinent to making therapeutic plans that are going to be more likely to succeed… I’m genuinely interested in who they [patients] are as individuals. I think that can be only expressed by having those types of conversations and asking the follow up questions: “so did you get in touch with your daughter, how’d that work out? How’s the housing situation coming along, any luck, any leads?"

At a minimum, people’s social circumstances and the restrictions that these circumstances place on health must be understood and acknowledged. Further, practical steps can be taken, as this provider explained:

… there’s a water cooler [in the waiting room] and sometimes there’s leftover food… and we’ve had toothbrushes and toothpaste, and samples of stuff because we recognize… Well, nobody has even two dollars to buy that stuff… And so I think there’s that level of recognition of the degree of need, and the poverty that [some are] living in. And it’s understood and not judged.

At an organizational level, structures and time allocation must support providers to address people’s socio-economic needs, either through partnership arrangements with other agencies or by creating a network of multi-disciplinary team members to whom patients can be referred. This requires reconsidering priorities given the local organizational context, as this administrator explained:

Well when you sit back and think about it, there’s a need for so many things that are non-medical that ultimately come to bear upon people’s medical conditions. Like housing, and like even education… and those kinds of things. And you know, we’re not able to pay enough attention to those particular areas.

While PHC organizations can acknowledge people’s circumstances, create networks, and facilitate access to the social determinants of health, ultimately broader social change is required to achieve health equity. Health care providers and health care organizations must participate in broader democratic processes and social advocacy, bringing their unique vantage points to bear on public decision-making.

## Conclusions

The key dimensions of equity-oriented care and 10 strategies discussed in this paper may be most optimally operationalized in the context of interdisciplinary teamwork, however, they also serve as health equity guidelines for organizations and providers working in various settings, including individual primary care practices. Although the data were generated from research with two Indigenous PHC clinics in one province in Canada, our ongoing research using the key dimensions and 10 strategies indicates that they have broader applicability in a range of PHC contexts. These strategies can form the basis of organizational-level interventions to promote equity, and represent viable and “politically possible” [57] (p. 90) ways of increasing the provision of more equitable, responsive, and respectful PHC services for Indigenous populations. These approaches should not be conceptualized as solely applicable to Indigenous populations; rather, they have the potential to the provision of high quality care across population groups, moving care closer to the fundamental principles of quality PHC services.

Further research is needed to better understand how these approaches and strategies might intersect and lead to improvements in the overall quality of care, such as: an improved match between people's needs and services, enhanced trust and engagement by patients, a shift from crisis-oriented care to continuity of care, and an increase in the use of community-based services with potential for decreasing hospital admissions, readmission rates, and emergency department use. Studies focusing on the impacts of equity-oriented care should compare a range of contexts to deepen understanding regarding the appropriateness and applicability of these strategies. Future research is also required to measure the impacts of equity-oriented care on staff and organizational practices, patient outcomes, and ultimately on population health.

## Abbreviations

CAC: Community advisory committee
PHC: Primary health care
TVIC: Trauma- and violence-informed care

## Footnotes

1 PHC is the principal vehicle for the delivery of health care at the most local level of a country's health system, and is the first level of contact for individuals, families and communities, constituting the first element of a continuing health care process.

2 Our use of the terms ‘marginalization’ or ‘marginalized’ in relation to Indigenous peoples recognizes that marginalization is entrenched in the history of relations between Indigenous peoples and the nation-state, resulting in a disproportionate burden of ill health and social suffering within Indigenous populations. Marginalized therefore refers to the conditions that continue to result in structural, social and health inequities. Our use of the term is also inclusive of the many strengths and capacities accumulated in the context of people’s history and circumstances, and people's agency, resistance and resilience.

3 Consistent with accepted terminology used in landmark international reports, we use the term Indigenous peoples to refer to the diversity of populations throughout the world. In Canada, over 1.4 million people of the total population of ~ 32.9 million (4.3 %) identify as Indigenous [119], and the term Indigenous peoples refers to the original inhabitants of the land [120], including First Nations, Métis, and Inuit people. The term Aboriginal is also commonly used, and the colonial term ‘Indian’ is still used in federal government policy documents (e.g., The Indian Act). Approximately 50 % of Indigenous peoples are registered with the federal Department of Indigenous and Northern Affairs, and are therefore considered ‘Status’ Indians (First Nations). Thirty percent are Métis, 15 % are unregistered First Nations, and 4 % are Inuit. There are currently 617 First Nations ‘bands’ or tribal groups in Canada representing over 50 cultural groups [119]. Approximately 78 % of Indigenous peoples live off-reserve [121].

4 In British Columbia (BC), Canada, for example, Indigenous children are 14 times more likely to be taken into the ‘care’ of the child welfare system than non-Indigenous children during their school-aged years [122].

5 In Canada, visible minority refers to persons who are identified according to Canada’s Employment Equity Act as "persons, other than Aboriginal peoples, who are non-Caucasian... or non-white in colour" [82].

6 Friendship Centres have formed in many Canadian cities under the auspices of the National Association of Friendship Centres. Initially, these Centres were developed to provide supports related to employment, housing, health and education. Over the decades, the number of Centres and their range of services have increased significantly.

7 Culturalism is a form of stereotyping whereby culture, defined very narrowly and often in stereotypical ways, becomes the primary explanation for why certain groups of people may be experiencing particular health or social issues. Research continues to show that healthcare professionals frequently attribute people’s social problems to their cultural characteristics, for example, leading them to wrongly assume that violence toward women may be acceptable in particular cultural groups, or that some people are more prone to using drugs or alcohol because of ‘their culture’.

8 Smudging is a practice common to many Indigenous groups in North America. It usually involves ceremony and using the smoke of sacred herbs, grasses or tree branches to cleanse one’s self and thoughts.
